# Synergetic Effects of Hybrid Carbon Nanostructured Counter Electrodes for Dye-Sensitized Solar Cells: A Review

**DOI:** 10.3390/ma13122779

**Published:** 2020-06-19

**Authors:** Manas R. Samantaray, Abhay Kumar Mondal, Govindhasamy Murugadoss, Sudhagar Pitchaimuthu, Santanu Das, Raihana Bahru, Mohd Ambri Mohamed

**Affiliations:** 1 Department of Ceramic Engineering, Indian Institute of Technology, Banaras Hindu University, Varanasi, Uttar Pradesh 221005, India; manas.ranjan160@gmail.com; 2 Department of Electrical Engineering and Computer Science, Indian Institute of Technology, Bhilai, Chhattisgarh 492015, India; 3 Institute of Microengineering and Nanoelectronics, Universiti Kebangsaan Malaysia, Bangi 43600, Malaysia; abhay.nano17@gmail.com (A.K.M.); raihanabahru@ukm.edu.my (R.B.); 4 Centre for Nanoscience and Nanotechnology, Sathyabama Institute of Science and Technology, Chennai, Tamilnadu 600119, India; murugadoss_g@yahoo.com; 5 Multifunctional Photocatalyst and Coatings Group, SPECIFIC, Materials Research Centre, College of Engineering, Swansea University, Swansea, Wales SA1 8EN, UK; s.pitchaimuthu@swansea.ac.uk

**Keywords:** carbon, counter electrode, DSSCs, efficiency, nanomaterials

## Abstract

This article provides an overview of the structural and physicochemical properties of stable carbon-based nanomaterials and their applications as counter electrodes (CEs) in dye-sensitized solar cells (DSSCs). The research community has long sought to harvest highly efficient third-generation DSSCs by developing carbon-based CEs, which are among the most important components of DSSCs. Since the initial introduction of DSSCs, Pt-based electrodes have been commonly used as CEs owing to their high-electrocatalytic activities, thus, accelerating the redox couple at the electrode/electrolyte interface to complete the circuit. However, Pt-based electrodes have several limitations due to their cost, abundance, complicated facility, and low corrosion resistance in a liquid electrolyte, which further restricts the large-area applications of DSSCs. Although carbon-based nanostructures showed the best potential to replace Pt-CE of DSSC, several new properties and characteristics of carbon-CE have been reported for future enhancements in this field. In this review, we discuss the detailed synthesis, properties, and performances of various carbonaceous materials proposed for DSSC-CE. These nano-carbon materials include carbon nanoparticles, activated carbon, carbon nanofibers, carbon nanotube, two-dimensional graphene, and hybrid carbon material composites. Among the CE materials currently available, carbon-carbon hybridized electrodes show the best performance efficiency (up to 10.05%) with a high fill factor (83%). Indeed, up to 8.23% improvements in cell efficiency may be achieved by a carbon-metal hybrid material under sun condition. This review then provides guidance on how to choose appropriate carbon nanomaterials to improve the performance of CEs used in DSSCs.

## 1. Introduction

Human activities strongly depend on the abundance of natural resources, especially water, minerals, and energy. Technological development and economic growth largely depend on the availability of amply energy sources. Among the different energy conversion technologies available, solar cells appear to be the best alternative because of their abundant resource and continuous energy supply [1]. Various solar cell conversion technologies with improved efficiency have been developed [2]. Silicon solar cells show high efficiency, but with the different energy-intensive process, single-junction devices have an efficiency of 31% by the Shockley-Quiesser limit. The reason is due to the bandgap of active material in the silicon solar cells, and production cost at this moment is the backbone for exited silicon solar cell technology. Thus, the commercial applications of this technology are limited [3]. New technologies, such as thin films and organic solar cells, show the advantages of transparency, lightweight, flexibility, and lower cost compared with silicon-based devices [4]. The third-generation solar cells are based on nanostructured materials, quantum dots, conducting polymers, and other advanced quantum phenomena, including up-converted solar cells. However, dye-sensitized solar cells (DSSCs) and perovskite solar cells (PSC) are widely accepted as highly efficient third-generation candidates [5,6]. DSSCs, in particular, have attracted extensive attention on account of their feasibility, viability, and a better outcome performance [5,6]. Nanoporous semiconductor-based DSSCs have sparked much interest in recent years because of their low production cost and ease of fabrication. The performance of a DSSC depends on the semiconductor material used in its fabrication, its morphology, the structure of sensitizing molecules, and the redox mediator used. Unfortunately, the interfacial recombination of electrons injected by the sensitizer with the cations of the dye molecule or redox couple limits the efficiency of DSSCs. DSSCs show high efficiencies of up to 13% under direct sunlight and 11% in indoor applications [5,7].

DSSCs are photoelectrochemical cells featuring reasonable efficiency [3], environmental sustainability, and cost-effectiveness. These solar cells usually consist of a wide-bandgap semiconducting material, such as a mesoporous TiO_2_ film, ZnO thin film, or Nb_2_O_5_ film, a sensitizer, a liquid electrolyte (mediator), and a solid-state hole-transporting material [8,9]. The liquid electrolyte in DSSCs is placed between the photoanode and counter electrode (CE) and excelled by high power conversion efficiencies [8]. In solid-state DSSCs, the liquid electrolyte is replaced by a solid-state hole-transporting material as a mediator [9]. However, the low efficiency of such a device hinders its further commercialization. Further optimization and improvement of each component of the cell are thus required to achieve the desired efficiency. A large amount of effort has been put into the evaluation of hole-transporting materials, photoanodes, dye molecules, and other stable photoactive layers of solar cells [8,9,10,11,12]. The performance of a device depends on the material development and fabrication methodology, as well as cell design. The saturation current density at thermal equilibrium, *J*_0_, is expressed in Equation (1):

(1) $$J_{0}=\frac{RT}{nFR_{ct}}$$

where *R* is the gas constant, *T* is the absolute temperature, *n* is the number of electrons transferred, and *F* is Faraday’s constant. This equation shows the importance of the charge transfer resistance (R_ct_) in evaluations of cell performance [10].

**Figure 1:**
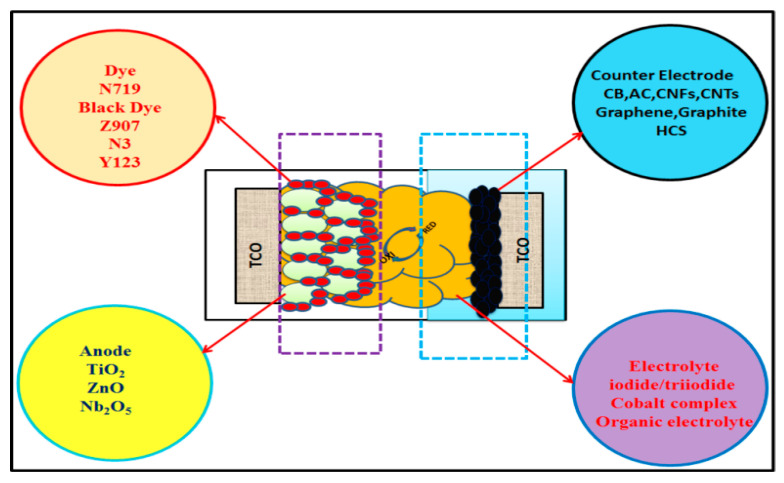
Schematic representation of the components used in dye-sensitized solar cells (DSSCs). TiO_2_ = titanium dioxide, ZnO = zinc oxide, Nb_2_O_5_ = niobium oxide, CB = carbon black, AC = activated carbon, and CNTs = carbon nanotubes.

A schematic diagram of the different components of DSSCs, as well as the different materials used in these components, is shown in Figure 1.

### Operating Principle of DSSCs

**Figure 2:**
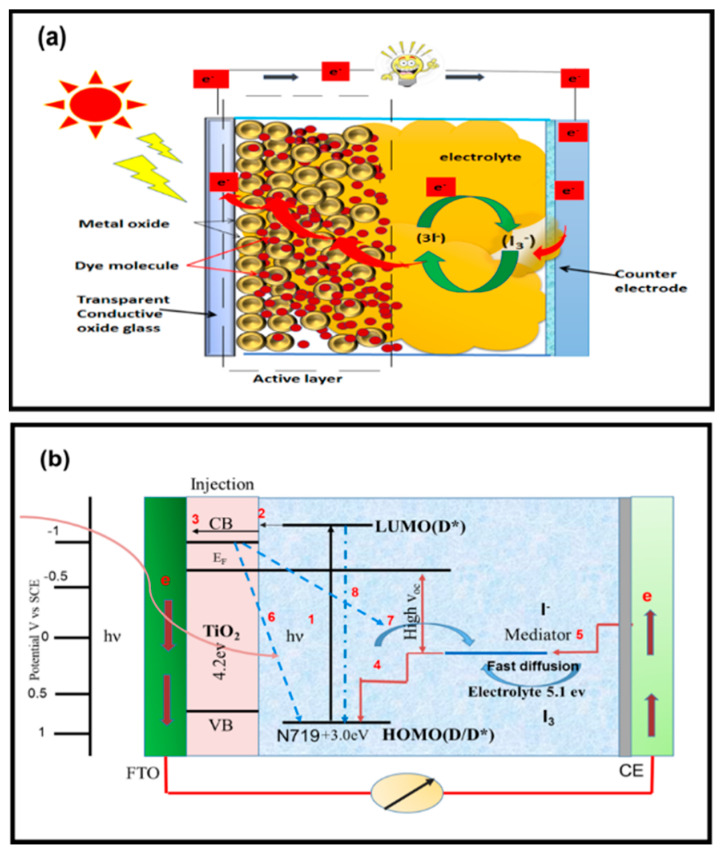
(**a**) Fundamental processes and constituent components of DSSCs. (**b**) Working principle and electron flow process of DSSCs.

DSSCs are composed of two electrodes sandwiched to form a third-generation solar cell device; they include four key components, namely, an anode, sensitizer, cathode, and electrolyte [13,14,15,16]. The performance of DSSCs depends on the electronic structure and morphology of the semiconductor material, the properties of the sensitizing dye, and the redox mediator (Figure 2a). The recombination of electrons with the holes of dye molecules at their interface region limits the performance and conversion efficiency of this device. During recombination, the dye absorbs photons and excite electrons to a higher state, which is denoted as (D*/Lowest unoccupied molecular orbital (LUMO)) (Figure 2b). A wide-bandgap semiconductor, such as TiO_2_, is used as a photoanode to evoke photoelectric effects [11,17]. After excitation, electrons from the highest occupied molecular orbital (HOMO) of dye molecules adsorbed on the surface of the photoanode are injected into the conduction band of the photoanode material. When the dye is reduced, the electron returns to its ground state, which allows the electron injection process to perpetuate. This process is facilitated by the redox couple, which is embedded in the electrolyte and in contact with the TiO_2_ layer. The “photohole” generated in the dye molecule is transferred to the electrolyte and oxidizes the redox couple. Thus, the redox reaction continues and leads to the formation of charge-carrier separation [18,19,20,21]. Processes 6 and 7 in Figure 2b explain the dark current reaction (charge-carrier recombination). This process dramatically affects the internal quantum efficiency, which refers to the conversion efficiency of the absorbed photon to the current generation in the external circuit [22]. Dark current reactions do not have a significant effect on the charge because of their slower reaction rate compared with that of current light reactions [23,24,25]. The lifetime of excited electrons plays a major role in the conversion efficiency of the device [21,23,24,25,26].

The main parameters affecting device performance include (i) the light absorption quality of the sensitized dye, where the absorption coefficient was in the range of 10^4^, while the others dye molecules have high absorption quality in the visible spectrum and (ii) the charge-carrier separation; development of these qualities is important to enhance the open-circuit voltage (V_oc_). The semiconductor morphology, light-harvesting efficiency, electron injection, and electron collection depend on film quality. Other parameters, such as electrical parameters, including series resistance (*R*_s_), should be very low (e.g., in the range of Ω/cm^2^). The shunt resistance of a device should be very high, and the optical properties of each layer must be enhanced. The light should reach a maximum absorption on the sensitized dye molecules [17,27,28].

**Figure 3:**
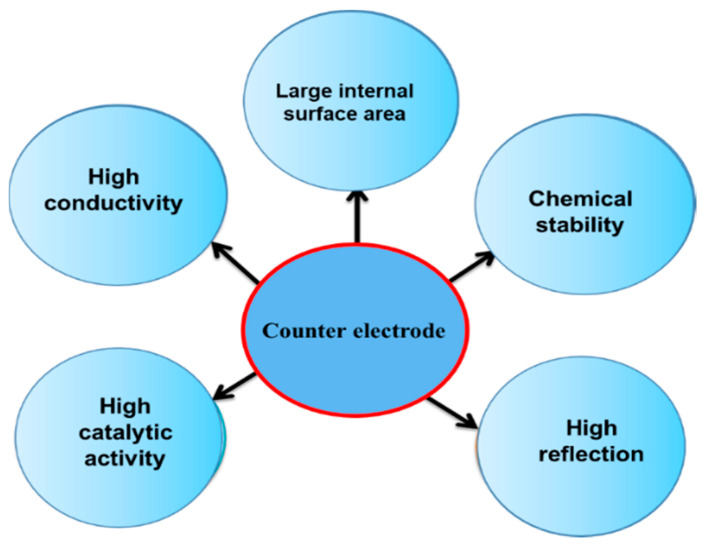
Properties required for high-performance counter electrodes.

Therefore, the performance of DSSCs relies on the electrode material and electrolyte. In this paper, an overview of the photoelectrode, electrolyte, and CE materials is provided. Many research groups are working in this emerging field of science to enhance device performance. As described earlier, DSSCs consists of three major active components. The CE influences the performance of the cell through its three major functions, namely, (i) catalyst, (ii) charge collector, and (iii) light reflector. (i) As a catalyst, a CE can improve the collection of electrons from the external circuit at the electrolyte/CE interface region to complete the cycle, i.e., the oxidized redox couple is reduced by the addition of electrons from the CE surface. For this mechanism to occur, a large surface area is needed at the interface region to create more active sites for reaction (Equation (1)). (ii) The CE must be a good conductor to allow the easy flow of electrons collected from the external circuit to the reaction sites. As a positive electrode, it extracts electrons from the external circuit and transfers them back to the cell. Ultimately, the function of CEs is to return electrons from the external circuit to the circulation path within the cell. (iii) As a mirror, CEs enhance the utilization of sunlight by reflecting unabsorbed light to the cell [23,25]. The properties of high-performance CEs are shown in Figure 3.

Carbon materials are abundant, low-cost materials with wide application prospects. Their industrial applications depend on several factors, among which ease of fabrication and material cost are the most important considerations. Carbon materials show impressive electrocatalytic activity on account of their multi-edge porous morphology, which provides active sites for the electrochemical reaction and high corrosion resistance toward liquid electrolytes. Hybrid carbon composites are emerging materials with dual characteristics. These carbon materials show potential use as CEs for DSSCs.

## 2. Counter Electrodes

The role of a CE is to collect electrons from the external circuit and reduce triiodide to iodide in the electrolyte. Ideally, the rate of electrolyte reduction at the CE should be comparable with the rate of dye regeneration by the electrolyte at the photoanode to maintain a low overvoltage and reduce energy loss in a DSSC, which is expressed by the short-circuit photocurrent density, J_sc_, in Equation (2) [25]:

(2) $$I_{3}^{-}+2e\to {3I}^{-}$$

**Figure 4:**
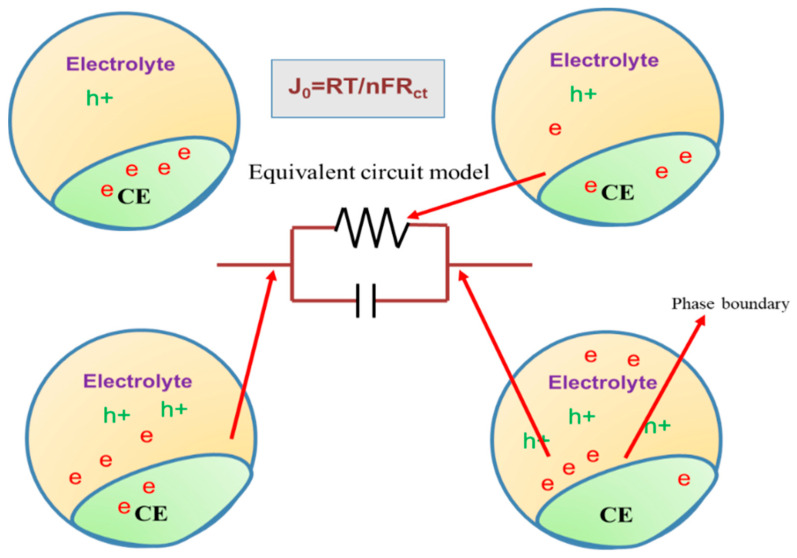
Schematic diagram of the reaction site at the counter electrode (CE)/electrolyte interface region and the electron transfer mechanism from the CE to the electrolyte material.

Equation (2) represents the cathodic redox reaction at the CE/electrolyte interface region. The redox couple is reduced by the addition of electrons from the CE surface. For this mechanism to occur, a large surface area is needed at the interface region to create more active sites for reaction (Equation (1)) [29]. The above discussion is schematically illustrated in Figure 4.

The performance of DSSCs significantly decreases when an inefficient CE is employed, which offers high resistance by slow reaction [15]. The R_ct_ of Pt is 2–3 Ω cm^2^ and has 80% transparency at the visible spectrum, which gives high cell performance [26,30]. The Pt was used as a CE because it has high catalytic activity, low R_ct_, and good conductivity as in the previous study [31]. Pt-CEs catalyze the reduction of tri-iodide (I^3−^), which is produced by the oxidation of iodide electrolytes by the dye at the CE/electrolyte interface. It is widely preferred as the active material in DSSCs and hence showed the second-highest percentage in DSSCs performance [32,33,34,35]. Although Pt can reflect on the wavelengths that are not initially absorbed by the dye, it is very expensive compared with other electrocatalysts. Moreover, Pt-CEs show poor resistance toward corrosion in iodide solution, which may result in the formation of PtI_4_ [36]. These challenges have motivated efforts to explore other low-cost materials with the lowest possible Rs and good catalytic activity for CEs [37], for utilization in both traditional iodide and new redox couples (T_2_/T^−^, Co^3+^/Co^2+^) [38,39,40]. Achieving all of these desirable qualities from a single-phase material is difficult because these qualities depend on different morphologies and material phases. Different advanced materials, such as graphene and other carbon materials, have been considered for application as a CE in DSSCs. Hybrid materials, composite materials, and carbon materials with different alloys are also favored by different research groups. In this review, we describe some of the different carbon materials and their morphologies and properties for use as CEs in DSSCs. The Pt and electronic properties of multiwall carbon nanotubes (MWCNTs) give advantages to catalytic activity. Kuan-Chieh et al. studied the dispersion of Pt nanomaterial on MWCNTs and the fabricated device showed an improved efficiency of 8.23% compared with that of a Pt-based CE photo conversion efficiency of 6.90%. Moreover, the device attributed to higher J_sc_ of 18.01 mAcm^−2^, which is higher compared to a Pt-based CE 14.62 mAcm^−2^ [41]. The scanning electron microscopy (SEM) analysis revealed that the composite CE has a large surface area and abundant active sites, which enhance its electrocatalytic activity.

Poudel et al. synthesized electrospun carbon nanofibers/Pt composite (ECN-Pt) CEs for DSSC applications using a chemical route. They obtained a power conversion efficiency of 8%, which is greater than that of ECNs (6.3%) and Pt (7%) alone [42]. Besides Pt-carbon nanofibers (CNFs), carbon black (CB), carbon nanotubes (CNTs), graphene, graphene oxides, and different structures, such as mesoporous carbon materials with different morphologies, have been prepared as a hybrid composite to enhance the efficiency of Pt-based DSSCs [43,44]. Several research reports concerning carbon nanostructures and their hybridization methods for application to DSSCs have been published.

## 3. Carbon-Based Counter Electrodes

**Figure 5:**
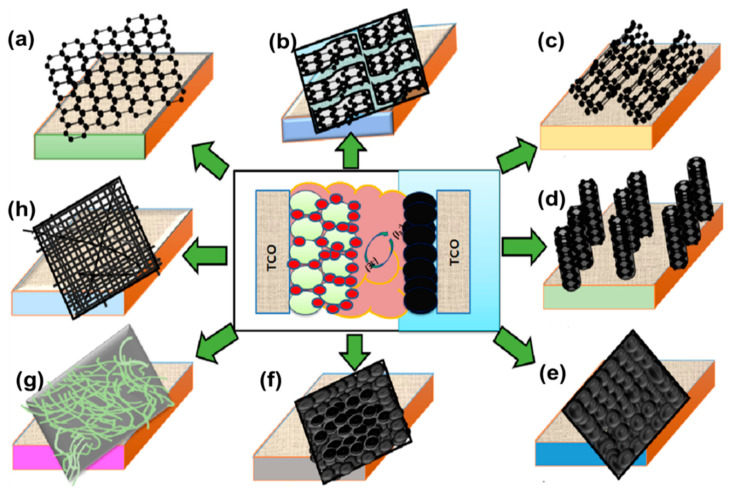
Schematic diagrams showing different types of carbon materials used as CEs in DSSCs: (**a**) graphene, (**b**) graphite, (**c**) randomly oriented carbon nanotubes (CNTs), (**d**) one-directional CNTs, (**e**) carbon black, (**f**) activated carbon, (**g**) carbon nanofibers (CNFs), and (**h**) hollow active CNFs.

Carbon is the most common material in the human and world. This element has several allotropes, namely diamond, graphite, C60 (buckminsterfullerene or buckyball), C70, amorphous carbon (activated carbon [AC], CB), CNTs, and graphene. Because carbon has desirable properties, such as high thermal stability, good corrosion resistance against liquid electrolytes, large surface area, high reactivity toward triiodide reduction, high conductivity, and catalytic activity, research on this material is abundant. Carbon materials are cost-effective, and graphene, CNTs, CNFs, AC, graphite, and CB, have been successfully employed as CEs (Figure 5) [45,46,47].

Among the carbon allotropes, graphene and CNTs are the most widely used because of their high mobility, which exceeds 200,000 cm^2^ V^−1^·s^−1^ at an electron density of 4 × 10^−9^ cm^−2^, electrocatalytic activity, and corrosion resistance toward iodide redox couples [48,49]. These desirable qualities enable the use of carbon allotropes as low-cost electrodes for DSSC and PSC technologies [50].

### Methods of Carbon-Based Counter Electrodes

Several synthesis methods for different allotropes of carbon can be used to obtain CEs for DSSCs, such as chemical reduction deposition, chemical vapor deposition (CVD), solution growth, heat pyrolysis, hydrothermal reaction, and sputter deposition. Table 1 shows different synthesis and fabrication methods to obtain carbon materials for CEs used in DSSCs. From this table, it is apparent that the carbon material synthesis and fabrication process significantly affects the performance of the carbon-based CE for DSSCs.

**Figure 6:**
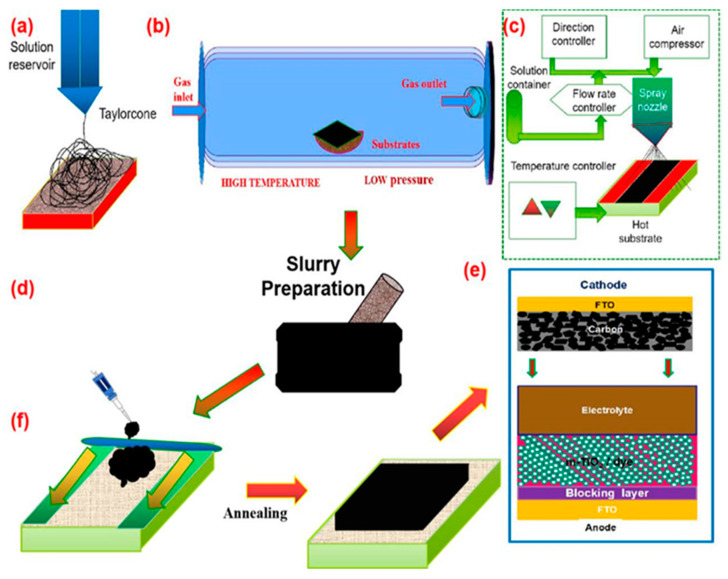
Schematic diagrams of different synthesis and fabrication methods of carbon materials used as CEs in DSSCs: (**a**) electrospinning method, (**b**) CVD method, (**c**) spray pyrolysis method, (**d**) slurry method, (**e**) CE fabrication method, and (**f**) doctor blade method.

These synthesis methods greatly influence the surface area and particle size of the resulting electrode and, hence, influence the performance of the final device in terms of its catalytic activity and electrical properties. Given the rapid development of carbon electrode materials in recent years, the methods used to prepare these materials have also been diversified. In this review, we describe some of the more popular methods of preparing carbon materials and CEs with high efficiency. In the electrospinning process, fiber structured carbon synthesis uses a solution precursor with a minimum concentration. The electrospinning setup includes a high-voltage power supply, solution reservoir syringe and tylenol cone nidel at different voltage applied pressure was changed to obtain CNF-like structures with different dimensions, as shown in Figure 6a. Low solution concentrations afford a mixture of beads and fibers. As the solution concentration increases, the shape of the bead’s changes from spherical to spindle-like, and, finally, uniform fibers with large diameters are formed because of high viscosity resistance. An optimum solution concentration must be established for the electrospinning process because low-concentration solutions yield beads instead of fibers and high-concentration solutions inhibit the formation of continuous fibers; in the latter case, the high flow of the solution at the tip of the needle cannot be maintained, and large fibers are obtained. Several researchers have attempted to determine the relationship between solution concentration and fiber diameter and found a power-law relationship in which the concentrations of the solution and fiber diameter concurrently increase during gelatin electrospinning. Solution surface tension and viscosity also play essential roles in determining the range of concentrations from which continuous fibers can be obtained during electrospinning. Prashant Poudel et al. synthesized CNFs using the electrospinning method [38].

Chemical vapour deposition(CVD) is a chemical process used to produce high-quality solid materials. In a typical CVD process, the substrate is exposed to one or more volatile precursors. The introduced gases then react and decompose on the substrate (Figure 6b) to achieve different parameters and structures of carbon allotropes. Nam et al. prepared aligned CNT arrays by CVD [55]. Spray pyrolysis is an aerosol process in which droplets are heated to break them down into solid particles as the precursor layer is in the range of nanometers. The nozzle system used for spraying follows a raster scan pattern. Veerappan et al. fabricated a DSSC with a spray-coated carbon CE and investigated the impact of the spraying time of the carbon paste on the performance of the resulting glass/plastic-coated carbon CEs. The carbon CE spray-coated on glass and plastic achieved a power conversion efficiency of greater than 6.0%, and the highest power conversion efficiency (6.2%) was obtained at a spraying time of 420 s [56].

The doctor blade technique is an easy method to fabricate different patterns of thin films. Here, the carbon powder material is prepared into a paste and coated on different substrates according to the size requirement, as shown in Figure 6d,f. This technique is broadly used to fabricate thin films on conducting glass substrates fluorine doped Tin Oxide (FTO) with a high surface area. Moreover, the techniques yields layer thicknesses that vary from 10 µm to 150 µm. Furthermore, the paste preparation and blade speed can be controlled easily. Many scholars have used this well-known technique to fabricate thin films for DSSCs. Carbon electrodes are annealed at temperatures of over 300–400 °C to increase the stability of the film toward the liquid electrolytes used for DSSCs. Hence, annealing is necessary for the use of carbon electrodes in DSSCs [60].

## 4. Carbon Black Nanoparticles and Hybridization

**Figure 7:**
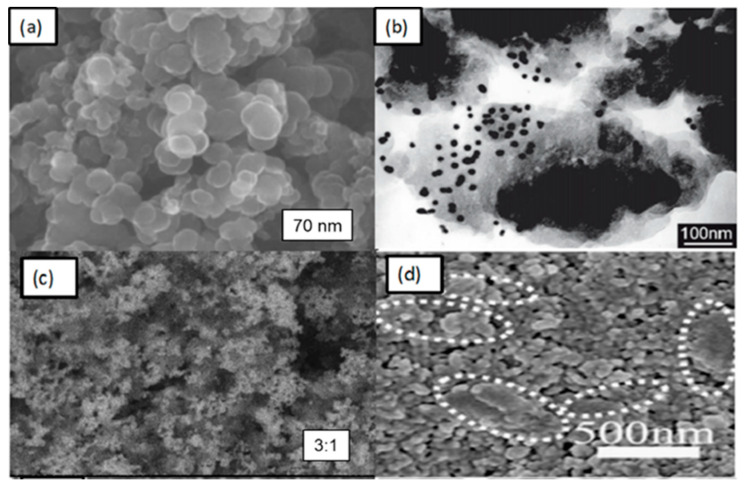
SEM morphologies of (**a**) CB and CB with different hybridizations, including (**b**) CB + Pt, (**c**) CB + TiN, (**d**) CB + Gr. Reprinted with permission from References [51,63,64,65].

Several researchers have reported the use of CB nanoparticles as CEs. Chen et al. and Joshi et al., for example, obtained efficiencies of 3.97% and 5.5%, respectively [49,61]. The properties of carbon materials, such as their morphology, particle size, the porosity of active sites, thickness, surface area, shape, and purity, play a vital role in enhancing the reduction process. In general, the thickness of the CB layer should be less than 10 µm to achieve good efficiency. The efficiency of CB could be improved by studying the relation between the layer thickness and R_ct_, similar to the work of Murakami et al. [62]. The work used primary parameters, such as fill factor (FF), V_oc_, and R_ct_, change according to the thickness of the carbon-coated CE which obtained a maximum cell efficiency of 9.1% under a sunlight intensity of 100 mWcm^−2^ (J_sc_ = 16.8 mA cm^−2^, V_oc_ = 789.8 mV, FF = 0.685). When the thickness of the film was increased to 14 µm, the device showed maximum efficiency. Kim et al. examined the effects of different particle sizes on CB with different thickness as a CE. They found that electrodes with small particle sizes and greater thickness produce better efficiency (7.2%) compared with a Pt-CE. Figure 7 shows scanning electron micrographs of the top view of different spherical CB nanoparticles [51] and their hybridizations. The micrographs demonstrate that spherical CB particles have large surface areas to accommodate redox reactions in CEs.

**Figure 8:**
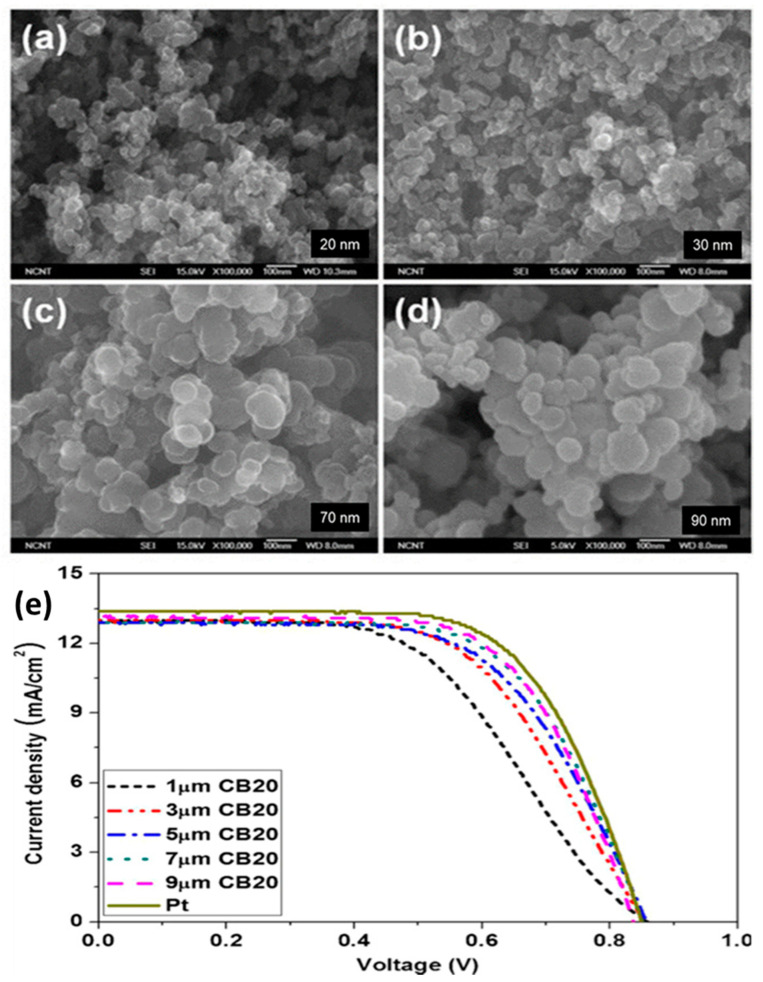
SEM morphologies of CB layers with various particle sizes of (**a**) 20, (**b**) 30, (**c**) 70, and (**d**) 90 nm, (**e**) Photocurrent-voltage curves for CB20 and Pt-CE-based DSSCs. Reprinted with permission from Reference [51].

Single materials show some favorable parameters, but hybrid materials give better performance because of their multi-functionality behaviors. Figure 7b–d showed the different morphologies of composite materials, such as (b) CB-Pt, (c) TiN-CB, (d) CB-Gr. CB spherical particles are uniformly dispersed in different carbonaceous materials and other matrices to obtain hybrid carbon composites. Figure 8 shows that increasing the particle size from 20 nm to 90 nm results in higher efficiency and lower R_ct_ due to the increase in surface area available for reaction sites. Figure 8b indicates that smaller particle sizes in the carbon CE significantly enhance the performance of the cell. Wu et al. [66] investigated the effect of the binder on the cell efficiency.

In a study by Wu et al., the complete removal of the binder yielded a maximum cell performance of up to 8.35%, which is higher compared with that achieved by a Pt-coated CE (8.29%). In addition, a nearly ideal R_ct_ was obtained from a CE without a binder when compared with that of an impure CB film. The corrosion resistance of the electrode material increases the stability and commercialization of DSSCs. Corrosion-resistant properties originate from the liquid electrolyte and are generally low for the iodide/tri-iodide redox couple. Hence, several alternative materials have been investigated as a replacement. The cobalt complex appeared to be well suited for this application because of its stability and cost-effectiveness. A CB CE with a Co (III)/(II) mediator was assessed by Yuh Lang-Lee et al. Here, a CB thin film-based solar cell was sensitized using an efficient dye, Dyenamo Red Y123. The device produced a conversion efficiency of 8.81% without using striking mass transport obstruction. The feasibility of CB as a CE was also verified [65]. Here, CB film was coated using a spin-coating method to a very precise thickness of ~1.4 µm. A Z907 sensitized cell with a minimum thickness of CB yielded a power conversion efficiency (PCE) of 7.21% and R_ct_~0.39 Ω cm^2^.

**Figure 9:**
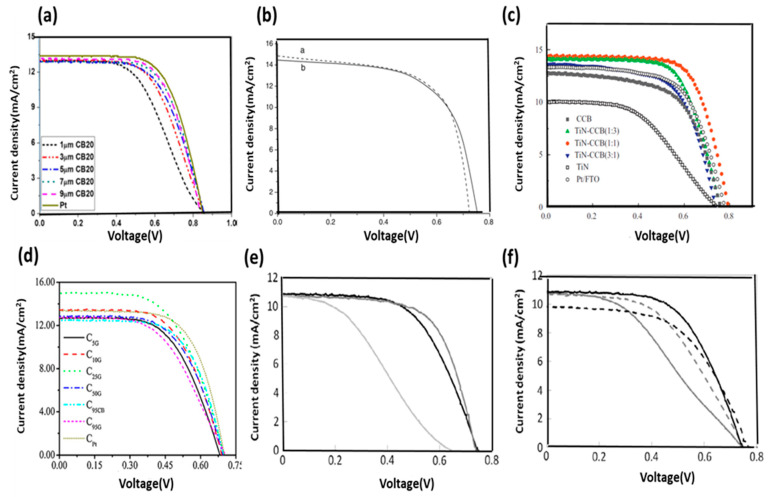
Characteristic J–V curves of hybrid carbon materials: (**a**) CB, (**b**) [CB + Pt], (**c**) [CB + TiN], (**d**) [CB + Gr], and (**e**,**f**) [CB + poly(3,4-ethylenedioxythiophene (PEDOT)]. Reprinted with permission from References [51,63,64,65,68].

However, thicker films could affect the fabrication of the cell and, in turn, decrease the stability of liquid-based DSSCs. A thick CB coating causes the CE to become opaque, which affects the latter’s transparency. Carbon materials generally have low intrinsic electrocatalytic activity [67]. Hybrid carbon materials have emerged as candidate materials for high-performance CEs. Figure 9 shows the cell performance of carbon CEs with different hybridizations, including (a) CB, (b) [CB + Pt], (c) [CB + TiN], (d) [CB + Gr], and(e), (f) [CB + poly(3,4-ethylenedioxythiophene (PEDOT)]. The figure clearly shows that solar conversion efficiency is greatly improved with hybridization (Figure 9).

P. Li et al. studied the hybrid composite of [CB + Pt] and showed an efficiency of 6.72% with a Pt weight loading percentage of 1.5%. The efficiency is higher compare to 100% of Pt loading and 0% loading [CB + Pt], the efficiency obtained was 6.63% and 3.76%, respectively. Similarly, the hybrid [TiN + CB] composite-based CE showed an increment of 7.17% efficiency compared with those of a Pt- and CB-based devices [TiN-CB]. C25G yielded a peak photoelectrical conversion efficiency of 5.99%, which is comparable with that of the single material-based [CB + Gr] device. A 0.5 wt % CB-based CE showed the highest efficiency of 4.01%, which is greater than that of a single material-based device [PEDOT + CB].

Photovoltaic parameters of one dimensional and two-dimensional carbon materials showed a significant improvement in photocurrent density of DSSC, as listed in Table 2. The hybridization of low-cost carbon counter electrodes is emerging for large-area modules. Thus, the low cost and high stability of carbon materials are essential solution for the commercialization of DSSCs. Among all conducting polymer composites, poly (3,4-etylenedioxythiophene) polystyrene sulfonate (PEDOT:PSS) performs better because the long chain in the negatively charged PSS alkali hinders the movement of I^3−^ ion towards the active site of positive PEDOT molecules. The charge transfer resistance (R_ct_) of the graphene/PEDOT:PSS counter electrode is low and showed high catalytic activity with high transmittance. Several possibilities of composites of different conducting polymers and their composite with carbon materials are selected from this table.

A relatively large-area module based on carbon CEs with a lifetime up to 10,000 h was recently reported. Thus, the low cost and high stability of carbon materials render them an essential solution for the commercialization of DSSCs.

## 5. Activated Carbon and Hybridized Activated Carbon

**Figure 10:**
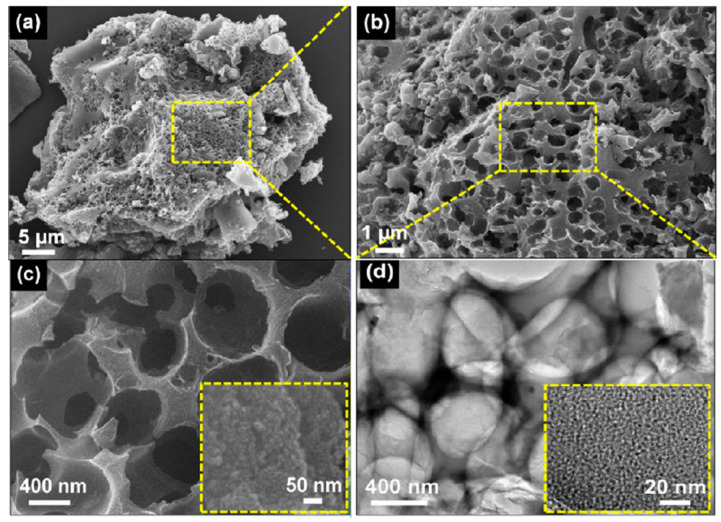
(**a**–**c**) shows scanning electron microscope (SEM) and (**d**) transmission electron microscope (TEM) images of a carbonized honeycomb porous carbon (HPC) sample under an inert gas atmosphere. Reprinted with permission from Reference [76].

AC has recently been paid increased attention by several research groups. AC has a highly porous structure (honeycomb-like) and enhances the catalytic activity at interface layers. The porous structure of the material acts as active sites for the electrochemical reaction. Figure 10a–c shows scanning electron microscope (SEM) images, while Figure 10d shows a transmission electron microscope (TEM) image of honeycomb porous carbon (HPC) [76].

**Figure 11:**
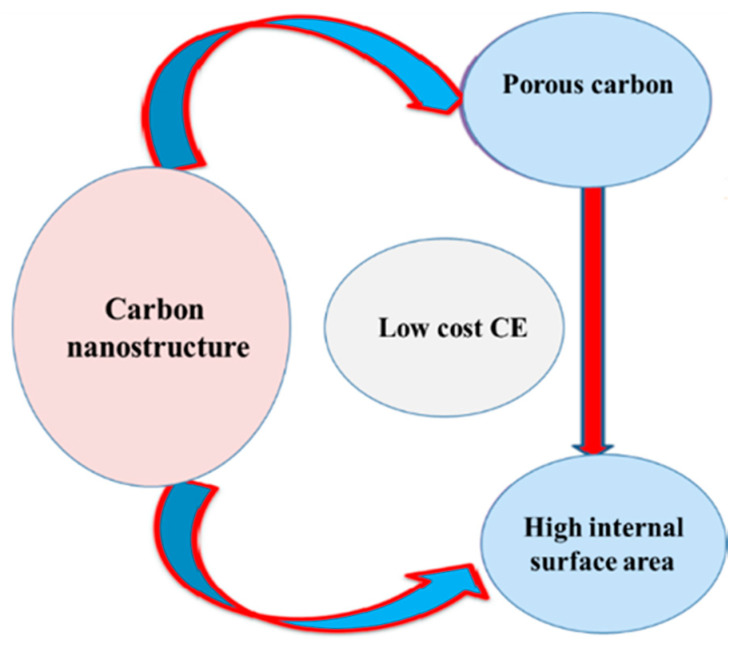
Schematic diagram showing the importance of porous carbon nanostructures as high-efficiency CEs in DSSCs.

A HPC-based CE could exhibit a PCE efficiency of 4.98%, which is higher than that of commercial AC-based CEs (4.45%) [76]. HPC is inexpensive, easy to synthesize, and possesses better adhesion to FTO substrates than AC; thus, the former is an attractive alternative CE material [76]. MM Ramil and LFA Talip used a composite of bamboo charcoal and TiO_2_ to fabricate a CE and obtained a high V_oc_ of 0.8 but a low efficiency of approximately 1.00% [77]. The attributes of different porous carbon nanostructures are shown in Figure 11.

**Figure 12:**
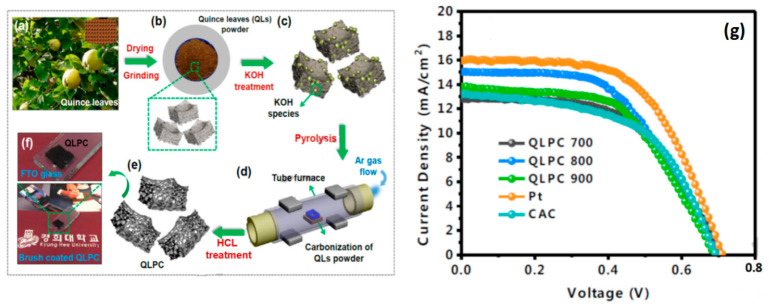
(**a**–**e**) Preparation procedure of quince leaves carbon powder (QLPCs) from QL bio-waste and (**f**) photograph of brush-coated QLPC on fluorine-doped tin oxide (FTO) glass for metal-free CEs in DSSCs. (**g**) Characteristic J–V curves of activated carbon QLPC annealed at QLPC temperatures of 700 °C, 800 °C, 900 °C or Pt-CE-based DSSCs. Reprinted with permission from Reference [52].

Different types of biowastes can be used as CEs in efforts to fabricate low-cost DSSCs compared with those requiring expensive metals, such as Pt [78,79]. AC has a large surface area that helps increase the availability of reaction sites. Activators such as KOH and NaOH are used to produce highly porous AC. The activation process is usually followed by the pyrolysis process, as shown in Figure 12a–e, and the resulting paste is coated on FTO substrates by using a brush coating technique (Figure 12f) [52,80]. AC resources are abundant because most biowaste carbon could be activated to produce porous AC. Among the various structures of activated carbon currently available, the honeycomb, flower, and three-dimensional structures are notable because of their large surface area [51]. The photovoltaic performance of AC-based CEs is shown in Figure 12g. DSSCs with AC CEs show V_oc_= 0.70 V, J_sc_ = 14.99 mA/cm^2^, and FF = 52.59%; they also have a higher efficiency of 5.52% compared with DSSCs with Pt-based CEs. The low electron transfer kinetics of mesoporous AC materials results in their low FF.

Mesoporous carbon materials have been considered potential CE materials because of their multi-edge porous morphology, which offers a large number of effective active sites for the electrochemical reaction; these materials are also characterized by low cost and high corrosion resistance toward the liquid electrolyte. These qualities are unidirectional. Some allotropes of carbon have many limitations that hinder their application as CE materials for DSSCs. Some studies observed that the large porous area (1300 m^2^g^−1^) of carbon materials enhances the efficiency of 8.14%. Recently, K.D.M.S.P.K. Kumarasinghe, et al. developed high conducting porous carbon material and fabricated high efficiency of 7.85% [81]. Lee et al. fabricated various carbon CEs with polyaromatic hydrocarbon film (LPAH) featuring a large surface area using different fabrication techniques [82]. LPAH particles with a uniform size of 10 nm thin film enhanced 45% of pore diameters for a specific surface area. The performance of the resulting material increased by 20.7% compared with that of a Pt/FTO CE substrate. Kumar et al. synthesized graphitic carbon by carbonizing sucrose and used the resultant material as a CE in DSSCs [83]. The performance of CEs is improved by fast electron transfer kinetics, high catalytic activity, and multifunctional behaviors, all of which could be achieved by hybrid nanostructured materials. Ma et al. recently reported on the performance of different types of AC electrodes and observed a high V_oc_ of up to 0.8 V [84].

## 6. Carbon Nanofibers and Hybridization as CEs for DSSC

**Figure 13:**
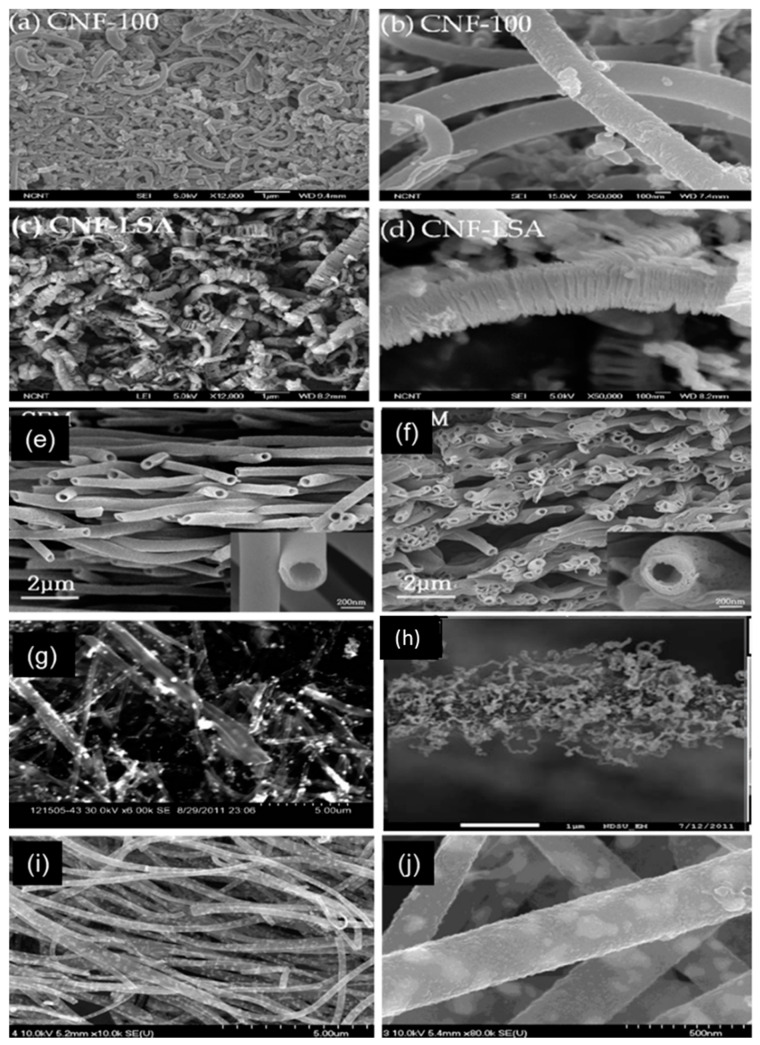
SEM surface morphology of carbon nanofiber (CNF) powders and films deposited on FTO glass substrates: (**a**) The CNF-100 film, (**b**) magnified image of the CNF-100 powder, (**c**) the antler carbon-nanofiber (CNF-LSA) film, and (**d**) magnified image of the CNF-LSA powder. Reprinted with permission from Reference [89]. (**e**) Cross-sectional SEM image of hollow activated carbon nanofibers (HACNF) and photocurrent-voltage curves (Figure 14) for HACNF, meso-HACNF1, and (**f**) meso-HACNF2. Reprinted with permission from Reference [57]. (**g**) Hybrid CNF-Pt. Reprinted with permission from Reference [42], (**h**) CNF-CNTs. Reprinted with permission from Reference [90], and (**i**,**j**) NiCo-NP-doped CNFs. Reprinted with permission from Reference [91].

Different research groups have studied one-dimensional carbon nanostructures. The structure and cost of CNFs make them attractive materials for application in nano optoelectronics and electron transport. CNFs are low-cost one-dimensional materials that are easier to synthesis compared with other one-dimensional materials. The structure of CNFs can be described as sp^2^ hybridization-based linear filament. These CNFs have one-dimensional fibrous structures with diameters between a few and several hundred nanometers, and lengths of up to several centimeters [71,85,86]. These carbon structures are highly graphitic and have different morphologies, such as cones, cups, and plates [87,88]. CNFs are a type of CNTs that are highly graphitic and discontinuous. This type of fiber has a lower R_ct_ compared with those of Pt-coated CEs because of its stacking morphology (Figure 13).

**Figure 14:**
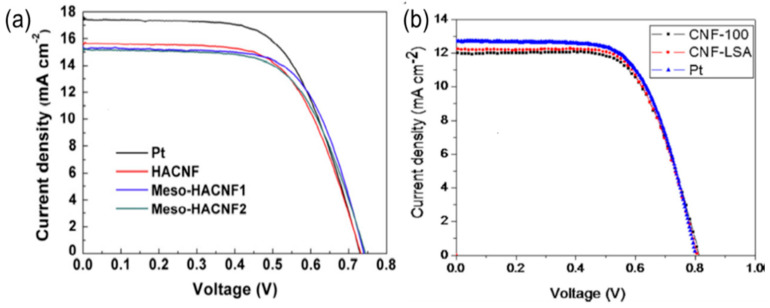
(**a**) Photocurrent–voltage curves for HACNFs, meso-HACNF1, meso-HACNF2, and Pt-based CEs. Reprinted with permission from Reference [57]. (**b**) Photocurrent–voltage curves for a quasi-solid-state electrolyte DSC with CNFs or Pt deposited on an Indium tin oxide coated polyethylene terephthalate (ITO/PET) flexible substrate as CE. Reprinted with permission from Reference [89].

CNF-based CEs with a film thickness of 12 µm have a low R_ct_ of 0.5 Ω cm^−2^ and cell performance of 7.00%, as shown in Figure 14a. The photovoltaic parameters of this type of CE are V_oc_ = 0.83 V, J_sc_ = 12.10 mA/cm^2^, and FF = 70.00%, which means it has a higher V_oc_ compared with other carbon-based solar cells. One-dimensional CNFs called hollow active CNFs (HACNFs) show a one-directional structure (Figure 13e,f) and a CNF-based CE shows higher efficiency compared with Pt-coated CEs (Figure 14b).

Joshi et al. investigated a novel type of ECNs as a CE for DSSCs. The CNFs were obtained by electrospinning using polyacrylonitrile (PAN) and then carbonized at 1200 °C in an inert atmosphere. ECNs were made into a paste using polyoxyethylene tridecyl ether as a binder, ground, and then sonicated. The resultant paste was made into a film, coated on an FTO substrate by using the doctor blade technique, and then sintered at 200 and 475 °C for 15 and 10 min, respectively [92]. The nanofibers showed the same adhesion problem as found in TCO glasses. In addition, the effective surface area increased with increasing layer thickness. However, thicker layers provided bulk resistance [89,93].

## 7. Carbon Nanotubes and Their Hybridizations as CEs for DSSC

**Figure 15:**
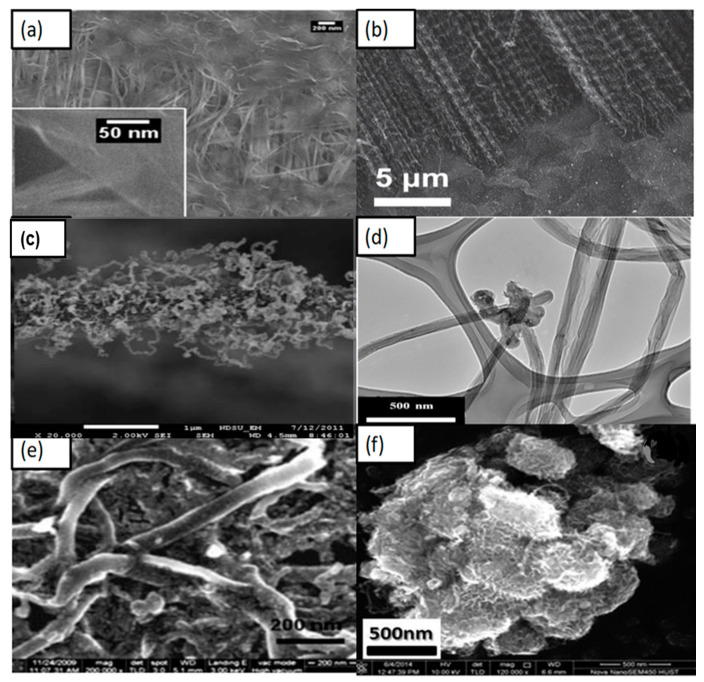
SEM morphologies of CNTs and CNTs with different hybridizations: (**a**) CNTs + Gr. Reprinted with permission from Reference [97], (**b**) Vertically aligned carbon nanotubes (VACNTs) + Gr. Reprinted with permission from Reference [98], (**c**) CNTs + CNFs. Reprinted with permission from Reference [90], (**d**) CNTs + rGo. Reprinted with permission from Reference [99], (**e**) CNTs + Pt. (Reprinted with permission from Reference [41], (Np)), and (**f**) CNTs + Ni. Reprinted with permission from Reference [69].

CEs require fast electron transport from the contact layer to the electrolyte. Among the many carbon materials studied for their catalytic activities, CNTs stand out because of their high catalytic quality and fast electron transport properties. CNTs are very fast in reducing reactions due to the one-directional path. CNTs are unique one-dimensional nanoscale structures with the combined advantages of large surface area, high conductivity, high mobility, high mean free path, and chemical stability. Two types of CNTs, namely, single walled carbon nanotubes (SWCNTs), which consist of a single roll of the graphene sheet, and multi walled carbon nanotubes (MWCNTs), which consist of several coaxial roles of graphene, have been developed for CEs. CNTs are highly suitable materials for nanoelectronic interconnectors because of their long mean free path (Figure 15a), which could encourage the flow of electrons. However, these materials do not show the same electrocatalytic activity as Pt [94,95]. MWCNTs with a bamboo-like structure show high efficiency and are suitable cathode materials for DSSCs. Different hybridizations with other carbon composites provide more defect-rich morphology for higher catalytic activity at reaction sites (Figure 15a–f). This CNTs structure has a large number of defect-rich edge planes, which ensures low R_ct_ at the interface between electrodes and electrolytes [96].

CNTs show poor adhesion toward TCO glass substrates on account of their weak Van der Waals bonding, which may affect their long-term stability risk. Ouyang et al. tested two types of CNT thin films with a binder and binder-free substrates where they found that *R*_s_ is low with the binder-free substrate and mentioned that nanotubes were covered by the binder and hence reduced the surface area [100].

**Figure 16:**
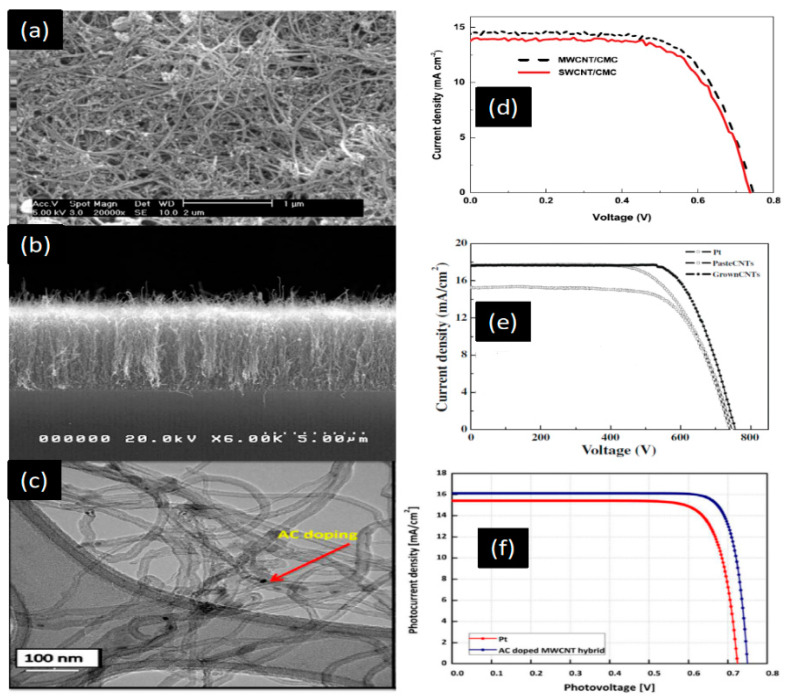
(**a**) SEM and (**b**) cross-sectional SEM image of CVD-grown CNT layer on a soda-lime glass. (**c**) TEM image of AC-doped MWCN composite, SWCNTs (**d**) I-V curve characteristics of SWCNT and MWCNT CEs for DSSCs. Reprinted with permission from Reference [54]. (**e**) I-V curves of DSSCs with different CEs. Reprinted with permission from Reference [101]. (**f**) Comparison of the photovoltaic performance of quasi-solid state DSSCs based on Pt and AC-doped MWCNT hybrid CEs. Reprinted with permission from Reference [59].

The authors reported R_ct_ = 0.6 Ω cm^2^ and an efficiency of η = 7.81%. SWCNTs show higher efficiency than MWCNTs (Table 3). MWCNTs are fabricated by CVD, as demonstrated by Nam et al. [101]. Well-aligned CNTs could be fabricated on FTO glass by using the CVD method (Figure 16b). The cell performance of well-aligned MWCNTs yields a PCE of 10.04%, which is higher than that of randomly oriented CNT films and Pt-coated FTO (Figure 16e). This higher efficiency is due to the high conductivity of one-directional CNTs. However, although CNTs have high conductivity and electrocatalytic activity, they are fairly expensive [102,103]. Table 3 shows the solar cell parameters and conversion efficiencies of some CE materials.

Table 3 listed the photovoltaic parameters of one dimensional and two-dimensional carbon materials and shows the influence of thickness towards charge transfer resistance that eventually improves the photocurrent efficiency of DSSC. From Table 3, it can be said that among these carbon materials, the AC + MWCNTs hybrid CE accomplished an efficiency of 10.05% with an optimum lower thickness of 3.0 µm. The detailed photovoltaic parameters such as R_ct_, V_oc_, J_sc_, and FF are displayed in Table 3.

**Figure 17:**
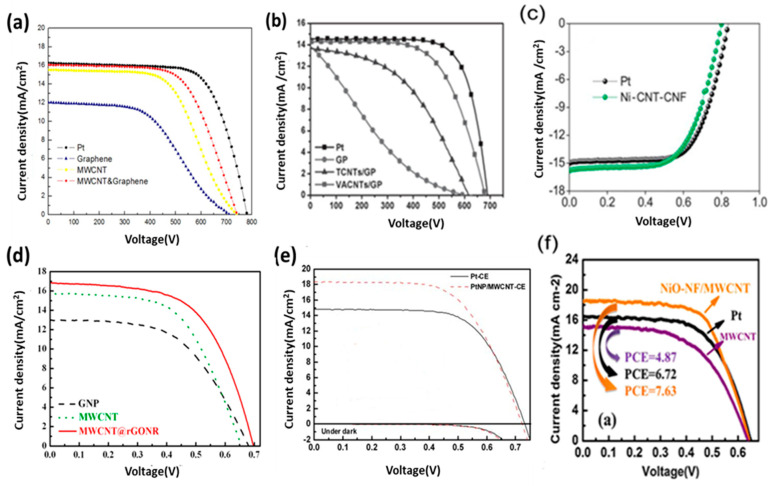
J–V curve of hybrid CNT materials: (**a**) CNTs-Gr. Reprinted with permission from Reference [97], (**b**) VRCNTs-Gr. Reprinted with permission from Reference [98], (**c**) CNTs-CNFs. Reprinted with permission from Reference [90], (**d**) CNTs-rGO. Reprinted with permission from Reference [99], (**e**) CNTs-Pt (Np). Reprinted with permission from Reference [41], and (**f**) CNTs-Ni. Reprinted with permission from Reference [69].

CNT paste prepared with a binder-free substrate shows higher efficiency compared with those prepared with cellulose and a Polyethylene glycol (PEG) binder. This higher efficiency is achieved due to the high conductivity of one-directional CNTs. Hybrid CNT composite materials show excellent photoconversion efficiency owing to their high catalytic activity and conductive nature, which is attributed to the one-dimensional nanotubes. In Figure 16, CNTs are uniformly dispersed with other materials to enhance cell performance. Highly conductive CNTs have high J_sc_ (Table 3). In 2016, Alvina Arban et al. carried out an experiment using MWCNT-dispersed AC as a CE for DSSCs, and obtained a high FF of 83% [59]. The researchers then demonstrated a new enzymatic synthesis route for highly conductive CNT dispersions (Figure 16c) [59]. Sung Hoonjeong et al. prepared an AC + MWCNT hybrid CE and obtained an efficiency of 10.05% and low internal R_ct_ of 0.60 Ω cm^2^ [59]. The authors used different enzymatic synthesis methods to enhance the efficiency of the device, and it showed that efficiency is higher due to the increase in the highest amount of FF up to 83%, which is obtained for DSSC application (Figure 16f). Electron conductive materials, such as MWCNTs, are used in composite, which determines the FF. MWCNT-doped microporous AC CEs strongly facilitate the fast release of I^−^ species when the electrolyte is reduced at the interface region and enhances V_oc_ [59,107]. Figure 16c shows a TEM image of the dispersion of AC with MWCNTs, but the conductivity of the carbon nanotube CE has to be improved. Other hybrids have emerged as potential CEs for DSSCs. The electrical conductivity of transition metals can be enhanced by controlling their structure. The efficiency of Ni_0.85_Se was observed by Jia et al. [108] to be 10.19%. Thus, Ni composites are attractive species for CEs in DSSCs. Joshi et al. synthesized a Ni-CNT-CNF material by electrospinning, mixing it with CMC binder and DI water, and then using the resulting material as a CE; the authors found a 7.89% efficiency under sunlight intensity (Figure 17c) [90]. Figure 16d–f shows the effect of hybridization of CNTs. Figure 17a–f illustrate that the photoelectrical conversion of hybrid composite CEs is higher than that of single-based CEs. Some improved photocurrent densities are shown in Figure 17d,e in which significant improvements can be seen in hybrid-based devices in comparison of single-based device.

## 8. Two-Dimensional Graphite/Graphene and Hybrid-Graphene Electrodes as CEs for DSSC

**Figure 18:**
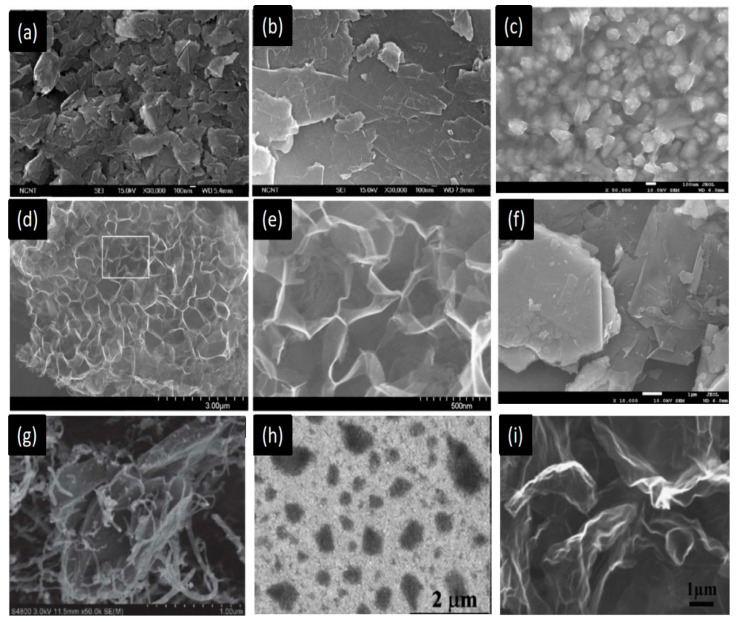
SEM images of (**a**) sub-micrometer- and (**b**) micrometer-scale graphite. Reprinted with permission from Reference [53]. FE-SEM surface images of (**c**) graphene/Pt hybrid. Reprinted with permission from Reference [118], (**d**) 3D graphene and (**e**) enlarged FE-SEM image of graphene. Reprinted with permission from Reference [55], (**f**) Gr-MoS_2._ Reprinted with permission from Reference [119], (**g**) Gr-CNTs. Reprinted with permission from Reference [56], (**h**) Gr-PEDOT. Reprinted with permission from Reference [120], and (**i**) N-Gr. Reprinted with permission from Reference [121].

Graphene is a two-dimensional nanomaterial with a very thin porous structure, large surface area (2600 m^2^g^−1^) [109] and has different allotropes, including graphite, CNTs, and fullerenes. Graphene also offers many advantages such as high electrical and thermal stability, as well as desirable optical and mechanical properties [47,110,111]. The material also has a high optical transparency of 97.7% [112], excellent thermal conductivity, high hole mobility, and abundance of oxygen vacancies/defects. These properties enhance its catalytic activity [113], corrosive resistance, low cost, and abundance [105]. Figure 18a–i show the different morphologies of graphene and its hybrids. Three-dimensional graphene materials have a large number of active sites for catalytic activities (Figure 18d,e).

**Figure 19:**
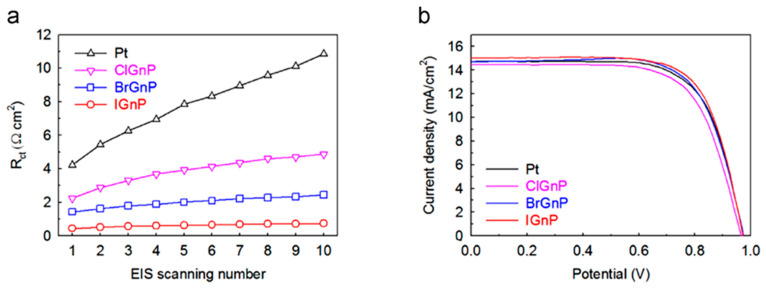
(**a**) Variations in the R_ct_ of Pt and XGnPs-CEs as a function of the EIS scan number. (**b**) J–V curves of CIGnP, BrGnP, IGnP, and Pt-CE-based DSSCs. Reprinted with permission from Reference [116].

These unique properties have recently given good performance in research for CE materials. Kaniyoor and Ramaprabhu showed that graphene has a R_ct_ as low as that of Pt [114]. Functionalized graphene sheets have been investigated as working CEs, and an efficiency of 10%, which is lower than that of Pt-based devices, has been obtained [115]. Jeon et al. demonstrated IGnP (iodine (I_2_) halogenated), CIGnP (chlorine (Cl_2_) halogenated), BrGnP (bromine (Br_2_) halogenated) materials as CEs. In particular, IGnP-CE offered a lower R_ct_ value of 0.46 Ω cm^2^ compared with that of Pt-based CEs (0.81 Ω cm^2^) under the same experimental conditions. This low Rct increased the FF by up to 71.3%, and, as a result, the PCE increased to 10.31% (Figure 19) [116]. Wang and Grätzel reported graphene-based CEs prepared using single layer-by-layer assembly with an electrochemical reduction procedure [34,117]. A thin layer of graphene, together with the heteroleptic Ru complex C106TBA as a sensitizer, was used as a CE to produce a PCE of 9.54%, which is higher than that of Pt-based CEs (9.14%). Thus, graphene exhibits promising potential as a substitute for the CE in DSSCs.

However, the production cost and use of toxic chemicals in conventional synthesis methods restrict the extensive applications of the material [122]. The challenges in the development of a graphene-based CE for DSSCs, including environmental stability, toxicity, and cost-effective production for commercial applications, has been reviewed [123].

**Figure 20:**
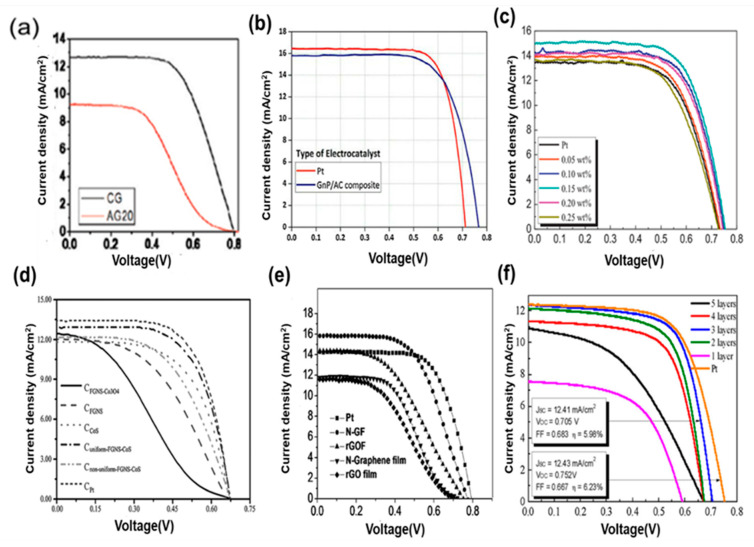
J–V curve of hybrid graphene materials: (**a**) graphite and A20 graphite, (**b**) graphite/activated carbon. Reprinted with permission from Reference [58]. (**c**) Gr + Pt. Reprinted with permission from Reference [118]. (**d**) Gr-CoS_2_. Reprinted with permission from Reference [124]. (**e**) nitrogen-doped graphene. Reprinted with permission from Reference [121] (**f**) Gr + MoS_2_. Reprinted with permission from Reference [119].

Some reports state that graphene-based PEDOT polymer matrices show high electrocatalytic activity. The R_ct_ of the graphene/PEDOT:PSS CE is low, and the device shows high catalytic activity. The PCE of hybrid CEs is approximately 7.86%, which is higher than that of Pt-CEs (7.31%). Hong et al. and Wu et al. respectively used spin coating and electrodeposition to deposit a thin film of graphene/PEDOT:PSS nanocomposite onto an ITO substrate [74,120]. PEDOT/Ex-Gr showed a highest efficiency of 8.0% under 100 mWcm^−2^ sunlight intensity of air mass (AM) 1.5 light, J_sc_ = 22.8 mA cm^−2^, V_oc_ = 640 mV, FF = 0.55. This device with a composite CE showed a high J_sc_ value of 22.8 mA cm^−2^ compared to that of individual cell structures. The development of two-dimensional transition metal dichalcogenide-based CEs and their hybridization with carbon-based CEs for DSSCs, instability challenges, and cost-effective production for commercial applications have also been reviewed. Most of the works are breaking through the limitations coming from single-material CEs in DSSCs because of its dual nature. Figure 20 shows the cell performance of carbon CEs and their different hybridizations. A hybrid composite of graphite/AC-based CE showed a maximum efficiency of 8.478% under an AC content of 60 wt %, as shown in Figure 2b; the efficiency obtained is better than that of the Pt-based CE (7.767%) [58]. Graphene/Pt showed 7.88% cell efficiency at 0.15 wt % Pt (Figure 20c). Figure 20d–f show composite materials named (d) Gr-CoS_2_, (e) nitrogen-doped graphene and (f) Gr + MoS_2_, respectively. The cell performance was improved due to their hybrid nature. A graphene/MoS_2_ hybrid composite showed a maximum efficiency of 5.98% and R_ct_ of 4.94 Ω/cm^2^, which is comparable with those of Pt-based devices.

A graphene/CoS_2_ CE was synthesized using a CVD method by Wonbong Choi et al. and found to be highly electrocatalytic toward iodine reduction with a low R_ct_ of 5.05 Ω cm^−2^. The enhanced performance observed was attributed to the increased number of active catalytic sites of G-CoS_2_ and the highly conductive paths of grapheme [124]. Jihuai Wu et al. fabricated a Ni_0.85_Se_0.15_/rGO film and used it as a CE; the material showed a maximum efficiency of 9.35% and low R_ct_ of 0.75 Ω cm^−2^ owing to its transparent nature [108].

## 9. Summary

In summary, DSSCs is an advanced third-generation solar cell. It stands as the rapidly developing research fields in this era for addressing various challenges regarding cost, stability, efficiency, scale-up production, etc. It is quite evident that developing DSSC while simultaneously addressing its major issues remain of interest for further development. The CE plays a vital role in deciding the cost and stability, at which site fast electron diffusion and electrochemical reaction influence the efficiency of the device. Some transition metals with a structure similar to platinum have been proposed to obtain good efficiency, but the corrosive nature of those materials towards liquid electrolyte is an undefined challenge. Some conducting polymers exhibited potential to be a good CE; PEDOT, for example, has good catalytic properties. However, the performance of the polymer CE-based DSSCs failed to reach the desired level of efficiency requirements. Ultimately, the carbon CE was introduced as low-cost material, which can be expected as a future material for low-cost, high-energy production devices.

Moreover, the thickness-dependent efficiency electrode which causes the opaque nature and affects the stability of the cell is a serious challenge. A material vs. PCE graph shows that carbon-carbon composite has the highest efficiency compared with metal-carbon and polymer–carbon composites.

Table 4 shows some published work on carbon CEs and their advantages, as well as some specific challenges to overcome for better performing device applications. Most of the nanostructured carbon materials such as carbon black and activated carbon are highly catalytic for a redox reaction, whereas these materials showed poor conductivity issues. These limitations could be overcome by synthesizing composites with different types of carbon allotropes. The table demonstrates the importance of the hybridization method in improving the properties of CEs by selecting suitable materials. Most of the collected materials have unique strengths, and their problems could coincide with other materials’ advantages. The most noticeable attributes of one-dimensional carbon counter electrodes (CNF and CNTs) and two-dimensional material graphene showed high conductivity features. Still, they faced several challenges; direct coating on the TCO substrate hindered in the case of CNTs and a graphene-based CE for DSSC. The hybridization of different composite becomes the solution, but several limitations, such as suitable methodology for composite mixing, composite stability, and device stability, are challenges from the application point of view.

As a comparison, the hybridization of proper materials enhanced the overall device performance. Example hybridization of 1D materials with low cost activated carbon overcame the processing cost and showed high efficiency-based devices reported to date was ~10% [59]. Two-dimensional graphene hybridization with low-cost materials showed a high efficiency of ~8.48 with low fabrication costs for mass production [58]. In our review, Table 4 indicates the future direction of the hybridization of carbon material as the CE in DSSC. Several limitations of the carbon counter electrode noted in Table 4 influence next-generation researchers to do the currently ongoing research in the field of low-cost carbon-based counter electrodes in DSSCs.

## 10. Challenges and Future Direction in the Hybrid Carbon Nanostructured CEs for DSSCs

Engineering for large areas with a proper selection of materials and their fabrication cost are challenges for future directional development of DSSC research. Designing DSSC to prevent leakage of liquid electrolytes and chemical stability is an ongoing research in the field for the future direction of large-area fabrication. The transmittance of the materials will be required for tandem solar cells. The development of graphene material for DSSCs could be the best solution for 2 terminal, and 4 terminal Tendam-based DSSCs. Indeed, the performance and application of hybrid carbon nanostructures exhibit a tremendous potential to enhance third-generation solar cell devices. Recent studies are focused on new hybridization for implementation in DSSCs, especially in CE.

However, several challenges must be overcome in the fabrication of CEs for DSSCs. For example, further exploration of other low-cost materials for CEs with fairly good catalytic activity is necessary. The third-generation emerging technologies are widely focused due to ease of fabrication and less material uses. In DSSCs, the fabrication cost is due to transparent electrode uses and both electrodes as solid films. The low cost of photoanodes is due to the availability of a large variety of TiO_2_ materials with good stability. By contrast, CEs are expensive because they employ expensive materials, such as Pt, metal oxides, and conducting polymers. The development of a carbon CE will be a game-changer in the field of large-area DSSC and PSC fabrication. There is a need for an improvement in cheaper and flexible electrodes for DSSCs. One of the primary benefits of carbon nanomaterial-based methods is compatibility with both flexible (metal or plastic) and inflexible (glass) cell substrates. Printable DSSCs are expected to suppress the future photovoltaic cell market and could provide various benefits, such as (i) very affordable and simple mass production; (ii) the ability to print on flexible substrates, such as plastics; (iii) a large surface area of the goal applications can be protected as a result of offering a larger area for photovoltaic harvesting; and (iv) the double for coloration and transparency, also enlarging its achievable applications. All of these factors will enable printed DSSCs to be tailored to suit various applications, including domestic and industrial roof surfaces, automotive vehicles, and indoor light-harvesting devices. Solving these major challenges will be useful for the vast application of large-area DSSCs.

Carbon material-based CEs show excellent R_ct_, but maintaining the optical transparency of the films presents a major challenge. The flexibility of low-cost carbon materials, such as CB and AC is a low-cost synthesis process, whereas the performance of the CE is not satisfactory in device application. Hybridized carbon CEs show better performance than conventional CE. In addition, the production process is reportedly carcinogenic and causes health problems. Among all the allotropes of carbon materials when the film was made from these materials, some of the materials, such as CNFs, are effectively required higher thickness for better performance. The shortcoming of cost-effective CNFs and graphene is that they have a wide scope for the application of the CE in DSSCs, whereas fabrication of thin-layer graphene to date was the biggest challenge for required substrates such as FTO.

The development of carbon counter electrode could be a game-changer in the field for large area DSSCs and other third-generation solar cell fabrications. Most of the nano-carbon materials are (i) low-cost materials and abundant in nature, and their (ii) flexibility (iii) and stability against corrosive chemicals make them an alternative counter electrode for emerging DSSCs application. The non-vacuum printable process is suitable for large-area fabrication processes. To date, this has been, and continues to be an ongoing research field, in which several challenges need to be solved; for example, compatibility with liquid solvents during the coating process and environmental issues related to CNT and graphene. Carbon films annealed at low temperatures showed low chemically stability towards liquid electrolytes; this was a limitation hindering the large-area application of carbon on both DSSCs and perovskite solar cells. Perovskite films are unstable at high temperature; this newly emerging research is ongoing in research fields regarding large-area carbon hole-transporting layers.

There are several peer-reviewed papers published directly related to the topic of state-of-the-art development of the carbon counter electrode for dye-sensitized solar cells. Among them, we have selected some publications that gave insights and guidance regarding new research in the field, and those that we believe will help spur interest among researchers. The research behind large-area fabrication of DSSCs requires suitable material to reach all desired qualities. Carbon counter electrode is an outstanding material that offers the advantages of a low production cost, a low processing cost, and good chemical stability for device operation. Therefore, some highlights points in this review would enhance the potential of this material in DSSC applications:

(1) The carbon CE and hybrid-based CE are comprehensively reviewed for DSSC application according to the recent study.
(2) The desired properties of a CE are briefly explained with emphasis on the importance of charge transfer resistance.
(3) Photovoltaic performance of various low-cost carbon-based counter electrodes and their composite as CEs for DSSC are tabulated.
(4) The various synthesis and fabrication techniques for a high-performance CE are also discussed.

## Figures and Tables

**Table 1:** Synthesis and fabrication methods of different carbon-based counter electrodes.

SL. No.	CE Material	Synthesis	Fabrication	Remark	Ref.
1	carbon black	Combustion of petroleum products.	Doctor blade	Conductivity is low	[51]
2	AC	Alkali treatment and pyrolysis process	Doctor blade	Conductivity is low	[52]
3	Graphite	-	Doctor blade	Transparent conductive oxide (TCO) free substrate can be possible	[53]
4	Carbon nanofiber (CNF)	electrospinning	Doctor blade	Required high thickness.	[42]
5	CNTs	Chemical vapor deposition (CVD)	Doctor blade	Stability is low	[54]
6	Graphene	CVD	Doctor blade	Coating on TCO is difficult	[55]
7	Reduced graphene oxide + CNTs	microwave-assisted reduction	Electrophoretic deposition	Mass production is possible but toxic process	[56]
8	Hollow activated-carbon nanofiber(HACNF)	concentric electrospinning	Spray-coating	High performance compared with CNF-based CE	[57]
9	Graphite + AC	-	a bar coating method	-	[58]
10	AC + multi walled carbon nanotubes (MWCNTs)	enzymatic dispersion	Doctor blade	High fill factor	[59]

**Table 2:** Photovoltaic parameters of DSSCs based on high-performance composites with different metal oxides and PEDOT-based composite CEs.

CE Material	V_oc_ (V)	J_sc_ (mA/cm^2^)	FF (%)	PCE (%)	Ref.
Pt + MWCNTs	0.74	18.91	62.00	8.23	[41]
NiO-Nf/MWCNTs	0.64	18.54	63.90	7.63	[69]
PEDOT + MWCNTs	0.72	17.00	66.01	8.08	[70]
Pt + CB	0.75	14.46	61.60	6.72	[63]
PEDOT/PSS + CB	0.76	10.80	57.00	4.70	[68]
NiO-Co-doped CNFs	0.74	11.12	54.00	4.47	[71]
Pt + CNFs	0.83	14.35	67.00	8.00	[42]
PEDOT + CNFs	0.72	13.96	65.19	7.16	[72]
Pt + GR	0.80	12.06	67.01	6.90	[73]
PEDOT/PSS + GR	0.77	15.70	65.00	7.86	[74]
PEDOT + EXGR	0.64	22.80	55.00	8.00	[75]

Charge transfer resistance, V_oc_ = open-circuit voltage, J_sc_ = short circuit photocurrent density, FF = fill factor, PCE = power conversion efficiency.

**Table 3:** Photovoltaic parameters of DSSCs based on carbonaceous CEs.

CE Material	Thicknes (µm)	R_ct_ (Ω/cm^2^)	V_oc_ (V)	J_sc_ (mA/cm^2^)	FF (%)	PCE (%)	Ref.
Carbon black	4.8	0.47	0.77	14.74	71.30	8.35	[66]
Carbon black (20 nm)	9.0	12.80	0.84	13.10	65.60	7.20	[51]
Carbon black	1.4	0.39	0.88	13.44	74.01	8.81	[104]
AC	12.0	25.90	0.70	14.99	52.59	5.52	[52]
AC coconut shell	52.0	1.58	0.65	19.49	62.00	7.85	[81]
AC + MWCNTs	3.0	0.60	0.76	16.07	83.00	10.05	[59]
Carbon nanofiber	12.0	0.50	0.83	12.10	70.00	7.00	[89]
Hollow active carbon nanofiber (HACNF)	1.6	5.40	0.73	15.40	64.00	7.21	[57]
SWCNTs	6.0	0.60	0.76	14.13	77.00	7.81	[54]
MWCNTs	6.0	0.75	0.74	14.49	71.00	7.63	[54]
Reduced graphene oxide	15.0	1.50	0.78	12.82	72.00	7.19	[75]
Honeycomb like structure graphene	20.0	20.00	0.77	27.2	37.00	7.80	[55]
Graphite carbon from sucrose	4–5.0	1.40	0.69	19.99	72.00	9.69	[83]
Graphite + AC	-	2.19	0.77	15.80	69.99	8.48	[58]
Graphite	9.0	5.00	0.79	12.40	61.00	6.01	[105]
Large surface polyaromatic hydrocarbon (LPAH)	3.0	2.12	0.80	11.50	80.00	8.63	[45]
Carbon + PEDOT	3.6	2.00	0.65	16.80	70.00	7.60	[106]

**Table 4:** Advantages and disadvantages of different carbon counter electrodes.

SL No.	CE Material	Advantages	Limitation	Challenges	Remark
1	carbon black	Lower cost than AC	Conductivity is low	Adhesion to the glass substrate	Performance is dependent on the film thickness
2	AC	Low cost	Conductivity is low	High temperature process	Composite with conductive material has high performance
3	Graphite	Low-charge transport resistance	Pure graphite showed a poor electrocatalytic ability	Methods for preparing paste	More suitable for large area fabrication technology
4	Carbon nanofiber	Flexible, lightweight	Larger dimensions compared to carbon nanotubes	Coating on glass substrate	Larger dimension limits the effective surface area, and as a result, a higher thickness is required
5	CNTs	1-D is a very high conductivity	Direct coating on substrate is difficult	Coating on substrate is difficult	Vertical coating on substrates showed high efficiency.
6	Graphene	Highly conductive	Fabrication cost is high	Coating on substrate is difficult	More suitable for flexible DSSCs and transparent DSSCs
7	Reduced graphene oxide	Low-cost mass production	Hazardous chemical process for the synthesis	A suitable method for preparing a film	Alternative for both large area solid and flexible substrates
8	Hollow active carbon nanofiber (HACNF)	High catalytic activity	Larger dimensions compared to carbon nanotubes	Preparation method cost is high compared to that of CNF	The larger dimension limits the effective surface area, and as a result, a higher thickness is required
9	Graphite + AC	High catalytic activity	Defect reaches surface to limit the charge transport process	Stability of composite	Solution preparation is easily compared to another composite
10	Graphite + CB	High catalytic activity	Less than 10% PCE	Suitable methodology for composite mixing	Low-cost high-efficiency counter electrode could be possible
11	AC + MWCNTs	High efficiency	Homogeneously mixed matrix	Stability	Composite mixing is a difficult process
